# Enhanced multiscale plant disease detection with the PYOLO model innovations

**DOI:** 10.1038/s41598-025-89034-9

**Published:** 2025-02-12

**Authors:** Yirong Wang, Yuhao Wang, Jiong Mu, Ghulam Raza Mustafa, Qianqian Wu, Ying Wang, Bi Zhao, Siyue Zhao

**Affiliations:** 1 College of Water Conservancy and Hydropower, Sichuan Agricultural University, Yaan, Sichuan China; 2 College of Information Engineering, Sichuan Agricultural University, Yaan, Sichuan China; 3 College of Life Science, Sichuan Agricultural University, Yaan, Sichuan China; 4 Shandong Baiyinnuo Biotechnology Co., Ltd, Zaozhuang, Shandong China; 5 Tea Research Institute, Yunnan Academy of Agricultural Sciences, Yunnan Key Laboratory of Tea Science, Kunming, 650205 China

**Keywords:** Plant disease detection, BiFPN, ECA, Feature fusion, Attention mechanism, YOLOv8n, Ecology, Evolution, Plant sciences

## Abstract

Timely detection of plant diseases is crucial for agricultural safety, product quality, and environmental protection. However, plant disease detection faces several challenges, including the diversity of plant disease scenarios and complex backgrounds. To address these issues, we propose a plant disease detection model named PYOLO. Firstly, the model enhances feature fusion capabilities by optimizing the PAN structure, introducing a weighted bidirectional feature pyramid network (BiFPN), and repeatedly fusing top and bottom scale features. Additionally, the model’s ability to focus on different parts of the image is improved by redesigning the EC2f structure and dynamically adjusting the convolutional kernel size to better capture features at various scales. Finally, the MHC2f mechanism is designed to enhance the model’s ability to perceive complex backgrounds and targets at different scales by utilizing its self-attention mechanism for parallel processing. Experiments demonstrate that the model’s mAP value increases by 4.1% compared to YOLOv8n, confirming its superiority in plant disease detection.

## Introduction

Plant diseases pose significant hazards, negatively impacting both agricultural production and ecosystems^1^. These diseases, caused by pathogenic microorganisms or abiotic factors, affect plant growth, development, and productivity, potentially leading to plant death in severe cases^2^. For instance, Panama disease has threatened the global banana industry since the mid-twentieth century, complicating banana production^3^. Similarly, coffee rust, caused by the fungus Hemileia vastatrix in 1970, has drastically reduced coffee yields and impoverished many farmers^4^. The likelihood of plant diseases is further increased by climate change^5^, failure of resistant varieties^6^, and the emergence of novel pathogens^7^. Therefore, developing a method for the timely and efficient detection of plant diseases is an urgent and critical task.

Previous research on plant disease detection can be categorized into traditional methods and computer vision methods^8,9^. Traditional methods involve physical and chemical sensors, as well as manual monitoring. For instance, early researchers used Polymerase Chain Reaction (PCR) to detect specific gene fragments of pathogens, thereby improving detection accuracy and specificity^10^. Additionally, hyperspectral cameras were employed to capture spectral information from plants, with changes in spectral characteristics analyzed to identify diseases^11^. Manual methods, such as symptom observation and microscopy, were also used for plant disease detection^12^. However, these traditional methods face significant challenges. PCR is prone to contamination, affecting accuracy, and is relatively expensive. Hyperspectral imaging techniques suffer from poor model generalization and are easily influenced by environmental factors like light, temperature, and humidity, which can interfere with data collection and analysis, compromising detection accuracy.

Manual monitoring demands substantial human resources and incurs high labor costs, leading to low detection efficiency. Consequently, computer vision methods, which facilitate timely and automated plant disease detection, have been widely adopted. Manual monitoring requires significant human resources and entails high labor costs, which often result in low detection efficiency^13,14^. These limitations make traditional methods inadequate for addressing the growing demands of plant disease management, particularly in large-scale agricultural settings. To overcome these challenges, computer vision techniques have emerged as a promising solution. By enabling timely, accurate, and automated detection of plant diseases, these methods not only reduce the reliance on manual labor but also improve overall monitoring efficiency^15^. As a result, computer vision-based approaches have gained widespread adoption in modern agriculture, revolutionizing how plant health is monitored and managed.

Research in computer vision can be categorized into traditional machine learning methods and deep learning approaches^16–20^. For instance, Marin et al.^21^ used decision tree-based modeling for mapping rust in coffee plantations, enabling effective, non-invasive disease surveillance. Abdulridha et al.^22^ utilized spectral features and multivariate analysis to detect wilt disease and nutrient deficiencies in avocado laurel, pinpointing the optimal detection band in the red, red-edge, and near-infrared spectra. Basavaiah et al.^23^ achieved 90% accuracy with a decision tree and 94% with a random forest classifier for tomato foliar disease classification. Hernández et al.^24^ employed Bayesian deep learning for plant disease detection, incorporating uncertainty quantification. They require significant manual input and extensive professional knowledge, often exhibiting poor model generalization. Consequently, deep learning methods, which can learn features autonomously, are increasingly used for plant disease detection.

Deep learning methods are primarily categorized into two-stage and single-stage approaches^25,26^. Two-stage deep learning methods include the R-CNN family of algorithms, such as the original R-CNN. These methods first generate candidate regions that may contain target objects through specific algorithms or mechanisms, followed by CNN classification and identification. This involves feature extraction and classification of candidate regions to determine the class of objects and optimize their location. Lin et al.^27^ utilized regional convolutional neural networks to develop a system for automatically identifying plant pests and diseases. Their results showed an accuracy of 89% for Faster R-CNN and 81% for Mask R-CNN in region recognition. Similarly, Bari et al.^28^ used Faster R-CNN to diagnose three rice leaf diseases (rice blast, brown spot, and leaf blast) with accuracies of 98.09%, 98.85%, and 99.17%, respectively, and healthy leaves with 99.25% accuracy. Dawod et al.^29^ used Faster R-CNN and Mask R-CNN for automatic lesion segmentation of diseases like Streptosporium and rust, achieving over 90% accuracy in segmenting at least one affected region per image with well-defined contours. While two-stage deep learning offers advantages in detection accuracy, it tends to affect detection speed due to the algorithm’s complexity. In contrast, single-stage deep learning methods are more adaptable and scalable, allowing for flexible adjustments and optimizations based on different tasks and datasets. As a result, single-stage deep learning has gained significant recognition as an important method for detecting plant diseases.

Single-stage deep learning methods include YOLO, SSD, and RetinaNet. Liu et al.^30^ used YOLOv3 to detect tomato plant diseases, enhancing speed and accuracy in image processing. Devisurya et al.^31^ improved YOLOv3-Tiny to boost detection accuracy and speed. YOLOv5 offers structural improvements over YOLOv3, providing higher accuracy and faster speed. Chen et al.^32^ introduced the Involution Bottleneck module and SE module in YOLOv5, achieving an average accuracy of 70%, 5.4% higher than the original. Wang et al.^33^ proposed an IASM mechanism based on YOLOv5, improving runtime and accuracy by 11.8% and 3.98% through the Ghostnet, WBF structure, and BiFPN.

Compared to YOLOv5, YOLOv7 employs faster convolutional operations and smaller models, resulting in higher detection speeds. Xia et al.^34^ enhanced the YOLOv7 lightweight model with MobileNeXt, achieving 93.5% accuracy, 89.9% recall, and 92.1% average precision, improving by 4.5%, 1.9%, and 2.6%. Umar et al.^35^ added SimAM and DAiAM to YOLOv7 for accurate field detection and classification of tomato leaves. These enhancements increased classifier accuracy to 98.8% and reduced the error rate to 1.2%. To further enhance detection accuracy and flexibility, YOLOv8 emerges as a superior option for plant disease detection due to technological advancements. Luo et al.^36^ proposed a lightweight self-attentive YOLOv8 model to improve detection accuracy across different scales. Uddin et al.^37^ employed an improved YOLOv8 model for automatic classification and localization, achieving precision of 93.2%, thus boosting agricultural productivity and sustainability. However, YOLOv8 requires substantial computational resources, has a long training time, and does not adequately account for small target classification and localization loss. Additionally, it needs enhancement in robustness to occlusion and rotation.

In response to these challenges, we propose a plant disease detection model named YOLO-ESC, which uses the lightest weight model, YOLOv8n, as the baseline. This model combines lightweight requirements with excellent detection accuracy. First, the weighted bi-directionally connected feature fusion mechanism (BiFPN) is used in the neck to address the multi-scale feature fusion problem in plant disease detection. Then, EC2f replaces C2f in the neck to efficiently capture the correlation between channel features, reducing complexity while enhancing feature fusion performance. Additionally, the newly designed MHC2f mechanism improves the network’s ability to characterize local features.

## Methods

### Structure of YOLOv8n

**Fig. 1:**
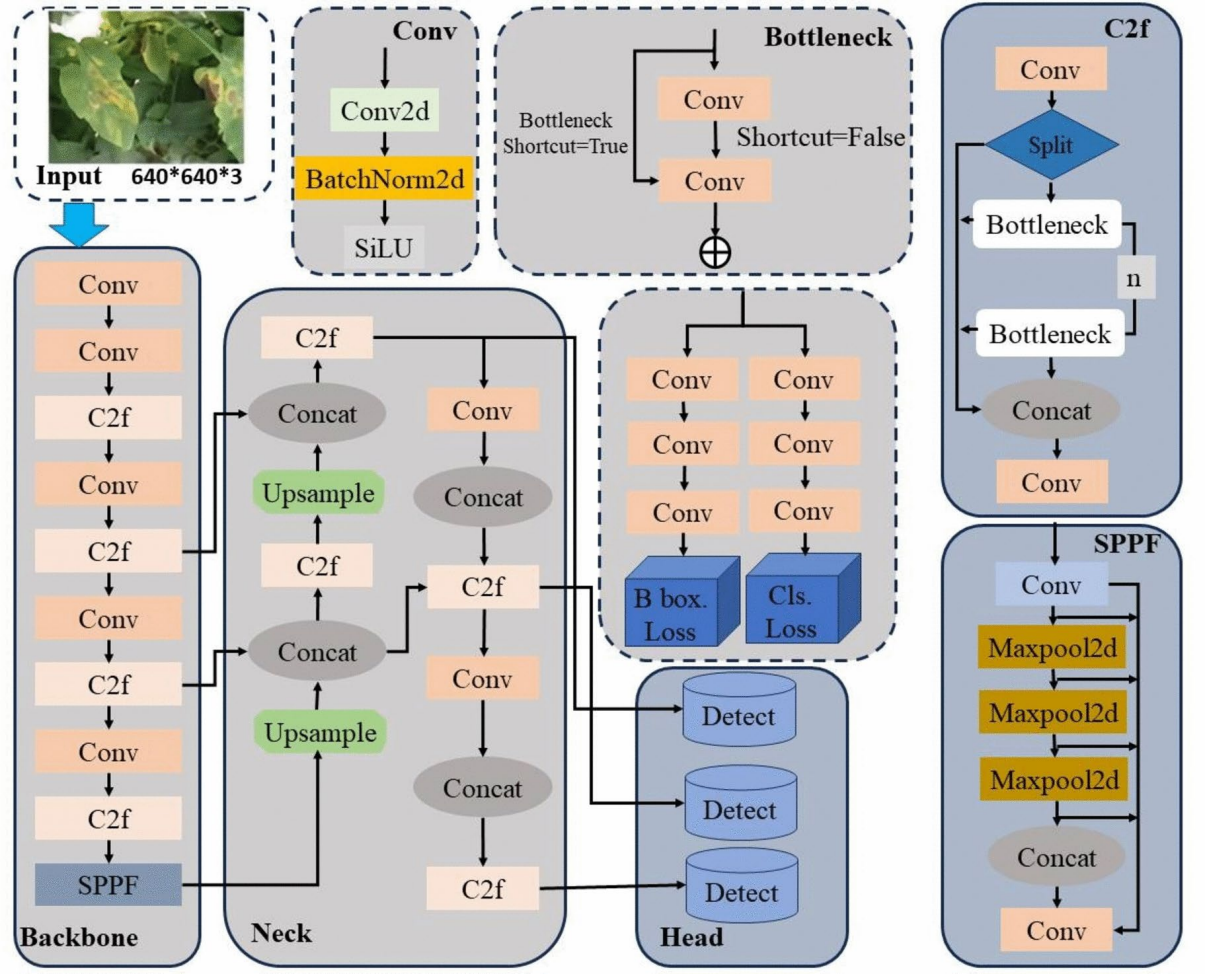
Structure overview of YOLOv8n.

YOLOv8, a leading model in the YOLO series, excels in target detection, instance segmentation, and image classification^38^. Its network structure consists of four parts: Input, Backbone, Neck, and Detection Head (Fig. 1).

Input: This stage primarily enhances data through the Mosaic technique, which increases the model’s robustness, simplifies model complexity, and improves accuracy via adaptive image scaling.

Backbone: Responsible for image feature extraction, this component comprises modules such as CBS, C2f, and SPPF. CBS performs convolution operations on the feature map, incorporating Conv, BN, and SiLU activation functions. YOLOv8 replaces the original C3 module in YOLOv5 with the new C2f module to capture rich gradient flow information. SPPF (Spatial Pyramid Pooling Fast) extracts features from different receptive fields to synthesize information at various scales.

Neck: This layer processes feature fusion for the extracted feature maps, primarily using the FPN-PAN structure. FPN employs a top-down approach for up-sampling, fusing higher layer feature information with lower layers. PAN introduces lateral connectivity to enhance the semantic information, allowing bottom-up feature maps to merge with top-down feature maps.

Detection Head: This component predicts the generated feature maps using an anchor-free matching mechanism to regress the target centroid and width-height at different scales, thereby reducing processing time. It leverages the rich information from feature maps at various scales to accurately obtain the classification and location of different target objects.

### Enhancements in YOLOv8n model

#### EC2f mechanism

**Fig. 2:**
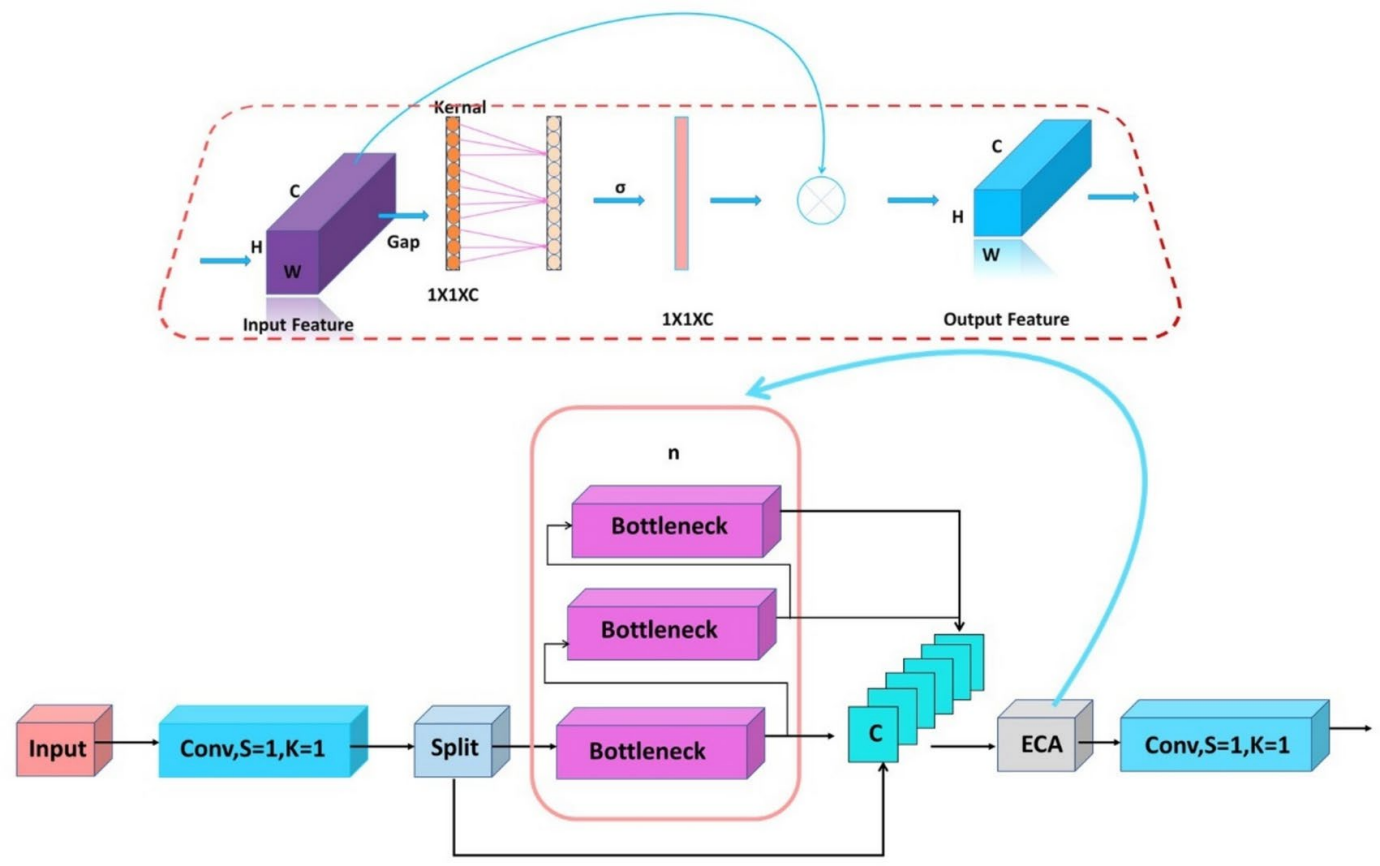
EC2f mechanism.

In plant disease detection applications, distinguishing disease features from other plant tissues in complex contexts is a significant challenge, often leading to false alarms. Although the C2f structure of YOLOv8 effectively captures local features through convolutional networks and improves feature representation by incorporating contextual information, it suffers from underutilization of output. To address this shortcoming, we propose an improved EC2f mechanism, as illustrated in Fig. 2.

The EC2f module enhances the traditional SE module by replacing the fully connected layer with a one-dimensional convolutional layer of adjustable kernel size. This design reduces model parameters and improves the CNN’s feature representation. By suppressing background features unrelated to plant diseases, the EC2f module minimizes background noise interference in target detection.

The process is as follows: The ECA module applies global average pooling (GAP) to the input feature map χ ∈ [h,w,c], yielding [1,1,c] feature vectors^39^. The main operation of GAP is the averaging of all the pixel values of each Feature Map, and finally, the output is a vector where each value corresponds to the global average of each channel. An adaptive 1D convolution kernel size k is computed based on the number of channels c. The 1D convolution output is then processed by the Sigmoid function to generate channel weights. These weights are multiplied with the original feature map to create a weighted feature map χ̅, enhancing relevant features for plant disease detection while suppressing irrelevant signals. Further details are provided below:

1 $$K = \left| {\frac{lb(C)}{\gamma } + \frac{b}{\gamma }} \right|_{{\text{odd }}}$$

where the parameter b adjusts the movement of the convolutional kernel size, while γ controls the rate of change of the kernel size concerning the number of channels.

#### MHC2f structure

**Fig. 3:**
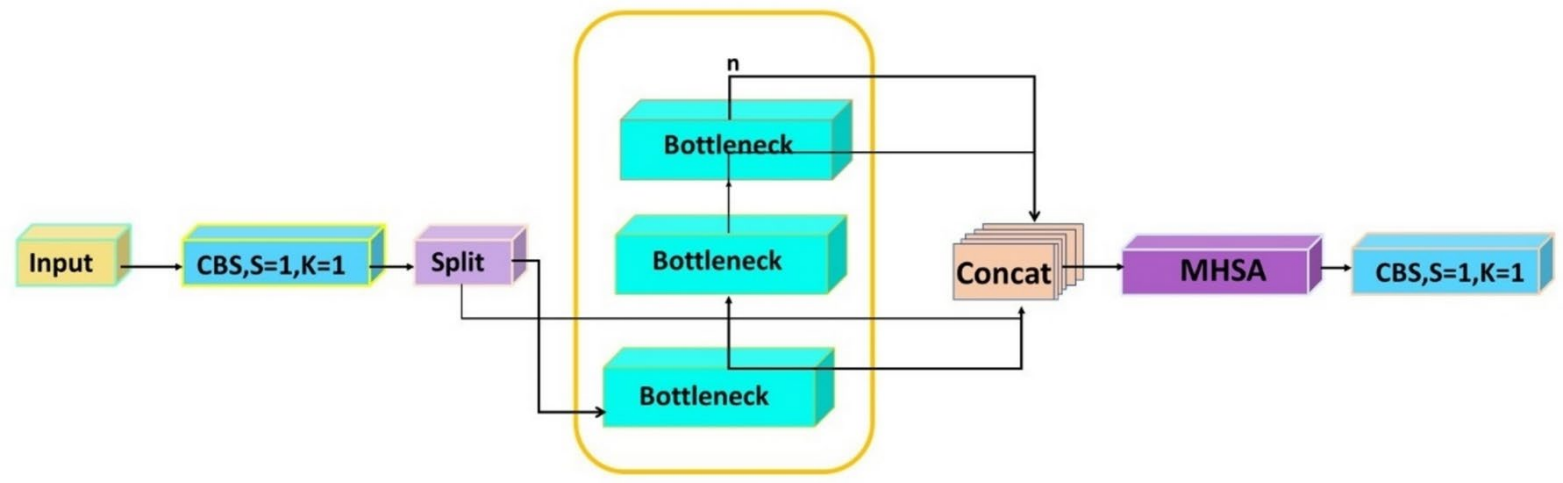
MHC2f architecture.

The challenge of plant disease detection lies in the variability of target sizes, significantly affecting diagnostic accuracy. To address this, we redesigned the MHC2f structure, as shown in Fig. 3. The MHC2f structure integrates strategies from the C2f model, which uses contextual information to enhance feature representation, and the Multihead Self-Attention (MHSA) model, which focuses on multiple spatial locations to facilitate information integration^40^. This approach not only enriches feature representation with contextual data but also enables the model to focus on multiple key regions in the image simultaneously. As a result, the model captures important details in complex scenes more effectively.

MHSA specifically enhances the model’s ability to focus on different image sections through parallel processing of the self-attention mechanism. This improvement significantly boosts the model’s capability to detect plant diseases and identify irregular tumor shapes at various scales. The accuracy and sensitivity of detecting plant disease features in complex environments have also improved. Additionally, the adoption of MHSA enhances the model’s computational efficiency, ensuring real-time detection requirements are met while maintaining high accuracy.

**Fig. 4:**
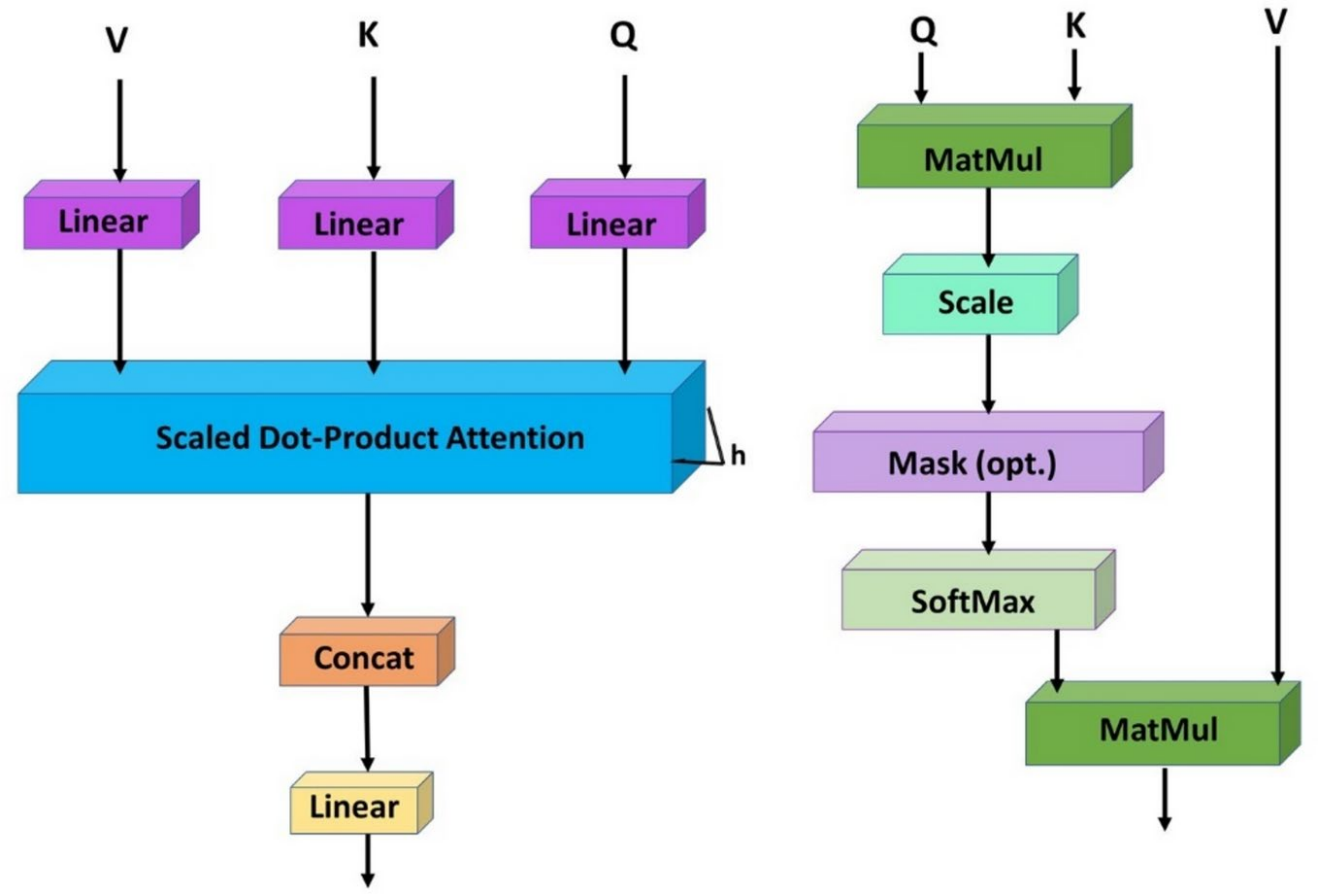
Incorporation of MHSA architecture.

MHSA operates on a core principle: by applying the attention mechanism to the same input multiple times in parallel, the model can capture different aspects of the relationship between inputs in various representation spaces. The core formulation of MHSA is divided into two parts: scaled dot product and multi-head attention, as illustrated in Fig. 4.

The scaling dot product mechanism computes the relevance of each key by calculating the dot product between the query (Q) and keys (K), then uses these weights for a weighted summation of the values (V) as follows:

2 $${\text{Attention}} (Q,K,V) = {\text{softmax}} \left( {\frac{{QK^{T} }}{{\sqrt {d_{k} } }}} \right)V$$

where the dimension of the key vectors is $d_{k}$. The scaling factor $\sqrt {d_{k} }$ is used to keep the dot product results manageable, ensuring the softmax function operates effectively. Without this scaling, large dot product values could push the softmax into regions with minimal gradients, reducing the efficiency of gradient descent.

Multihead Attention enhances this mechanism by projecting Q, K, and V into multiple subspaces via different linear transformations. It computes scaled dot product attention independently for each subspace, combines these results, and processes them through another linear transformation to generate the final output. This process can be summarized in the formula:

3 $${\text{MultiHead}} (Q,K,V) = {\text{Concat}} \left( {{\text{head}}_{1} , \ldots ,{\text{ head }}_{{\text{h}}} } \right)W^{O}$$

in this context, each ${\text{head}}_{{\text{i}}} = {\text{Attention}} \left( {QW_{i}^{Q} ,KW_{i}^{K} ,VW_{i}^{V} } \right)$, where $W_{i}^{Q}$,$W_{i}^{K}$ and $W_{i}^{V}$ are the linear transformation matrices for queries, keys, and values. The output linear transformation matrix enhances the input processing, leading to more robust output generation.

#### Bidirectional feature pyramid network

In plant disease detection, information about disease features at different scales is crucial for improving feature extraction and recognition. YOLOv8 handles targets with significant scale differences by introducing the PAN-FPN structure. This structure promotes information flow and fusion between high-level (low-resolution) and low-level (high-resolution) features, aiming to retain detailed information and enhance feature expressiveness. Although PAN-FPN improves the information fusion efficiency, it still faces challenges in capturing finer details. To further refine this process, this study employs a BiFPN^41^, which is an effective multilevel feature fusion strategy. The Bidirectional Feature Pyramid Network integrates both top-down and bottom-up feature flow paths and incorporates weighted contextual information edges, resulting in more detailed and efficient feature fusion.

**Fig. 5:**
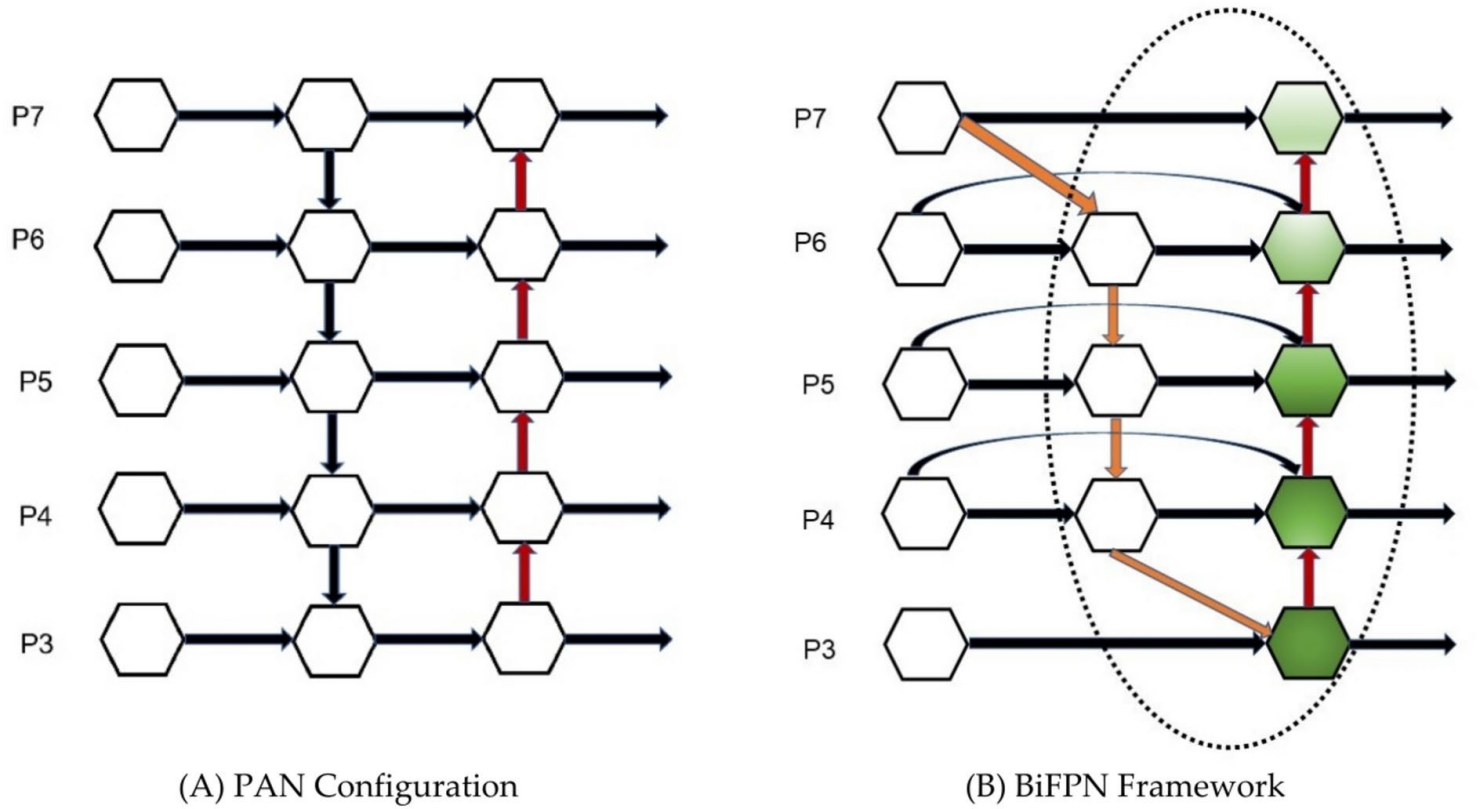
Comparison of feature fusion configurations. *Note*: (**A**) PAN Configuration: Illustrates the original Path Aggregation Network (PAN) configuration, highlighting its feature fusion pathway and semantic depth enhancement. (**B**) BiFPN Framework: Depicts the Bidirectional Feature Pyramid Network (BiFPN) framework, showcasing its optimized feature fusion with a weighting mechanism to reduce information loss and improve model performance.

This bi-directional feature fusion path not only deepens the semantic depth of the features, but also optimizes the contribution of different features through a weighting mechanism. This reduces information loss and improves model performance without significantly increasing the number of parameters. These improvements were visually illustrated in Fig. 5.

Compared to the original PANet, BiFPN improves feature extraction by removing less significant unilateral input nodes. When initial input nodes align with output nodes at the same network level, additional connections are introduced. By weighting and learning the importance of input features, BiFPN enhances detection accuracy. It processes bidirectional paths (top-down and bottom-up) in feature network layers, repeating this multiple times to achieve efficient bidirectional cross-scale feature fusion and fast normalized fusion.

Furthermore, BiFPN recognizes that the importance of different features varies significantly during the network learning process, and therefore adopts the strategy of assigning learnable weights to each input feature. These weights are learned adaptively by fast normalization, effectively solving the problem of resolution difference, which is generally ignored in traditional feature fusion methods. The weighting process, detailed in the provided formula:

4 $$O = \sum\limits_{i} {\frac{{w_{i} }}{{\varepsilon + \sum\limits_{j} {w_{j} } }}} \cdot I_{i}$$

where $O$ represents the output features, $w_{i}$ denotes node weights, and $I_{i}$ signifies input features. The learning rate $\upvarepsilon = 0.0001$ is set to ensure the stability of the values.

## Results

### Dataset related settings

**Fig. 6:**
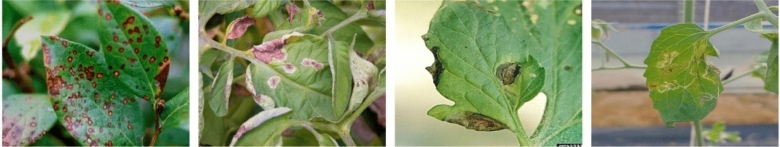
Some sample examples of plant disease.

Timely detection of plant diseases is crucial for ensuring agricultural production safety, improving the quality of agricultural products, and protecting the ecological environment. In this study, we used Google Maps and web crawling techniques to collect 10,000 images at a resolution of 1920 × 1080 pixels, creating a comprehensive dataset. Using the LabelImg image annotation software, we annotated nine target categories within the new dataset, with annotations stored in txt format. Examples of these annotations are provided in Fig. 6.

**Fig. 7:**
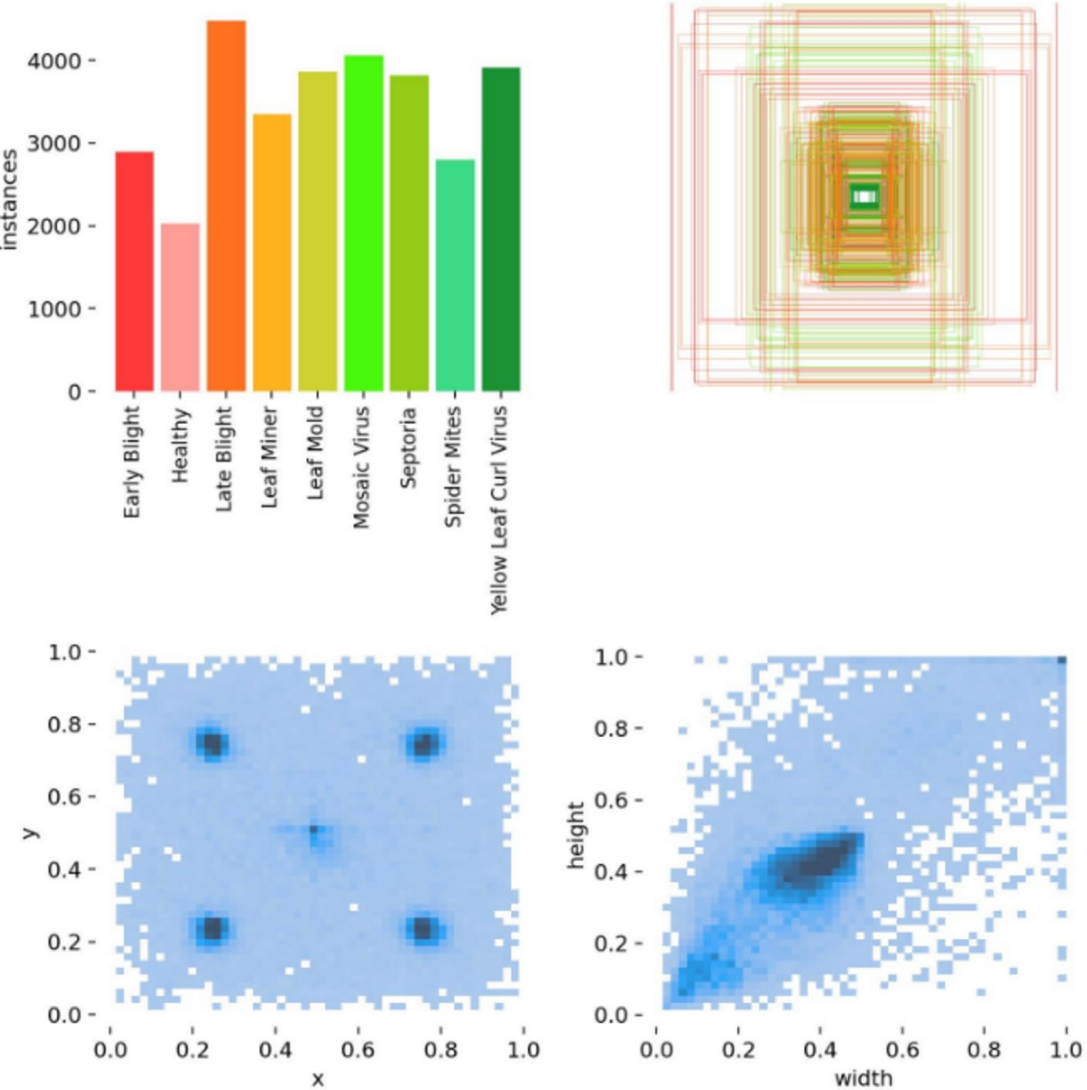
Analysis of plant disease data.

To meet experimental requirements, we randomly split the dataset into training, validation, and test sets in an 8:1:1 ratio. Figure 7 showed that the training set’s labels include the coordinates of the bounding box center point and the width and height distribution. The label file data format consists of five columns: label category, x-coordinate of the bounding box center point, y-coordinate, width, and height.

### Evaluation settings and metrics

The experiments were conducted on a Linux system using an NVIDIA GeForce RTX 4090 (24 GB VRAM) with PyTorch 1.7.0 and Python 3.8. Hyperparameters included an initial learning rate of 0.01, 300 training cycles, 0.937 momentum, 0.0005 weight decay, and a batch size of 32.

To evaluate the model’s effectiveness in detecting plant diseases, we used four key metrics: precision, recall, mean precision (mAP), and F1, as defined in Eqs. (5)–(8):

5 $$\begin{array}{*{20}l} {P = \frac{TP}{{TP + FP}}} \hfill \\ \end{array}$$

6 $$\begin{array}{*{20}l} {R = \frac{TP}{{TP + FN}}} \hfill \\ \end{array}$$

7 $$\begin{array}{*{20}l} {mAP = \frac{{\sum\nolimits_{q = 1}^{Q} A P(q)}}{Q}} \hfill \\ \end{array}$$

8 $$F1 \, = \frac{2*P*R \, }{{ \, P \, + \, R \, }}$$

precision, recall, mAP, and F1 are essential performance metrics. Precision is the proportion of true positives among the predicted positives. Recall is the proportion of true positives among all actual positives. mAP is the average precision at different recall levels, providing a comprehensive performance assessment. F1 is the harmonic mean of precision and recall, balancing both metrics. High precision and recall result in a high F1^42,43^.

### Pre and post-training evaluations

We conducted comparative experiments to demonstrate the superior plant disease detection capabilities of the proposed PYOLO model over the YOLOv8n sub-model. The results are shown in Table 1. These results indicated that the PYOLO model outperforms the YOLOv8n model with improvements of 6.7% in precision, 5.5% in recall, 6.1% in F1 score, and 4.1% in mAP. These gains result from three key enhancements: firstly, the innovative MHC2f module significantly improves the model’s ability to integrate features at different levels. Secondly, the EC2f module improves the efficiency of capturing effective information through feature weighting and tuning. Finally, the BiFPN module employs a progressive feature pyramid network to efficiently integrate multiscale features, which strengthens the model’s ability to recognize targets of different sizes.

**Table 1:** Effectiveness of plant disease detection.

Model	Precision (%)	Recall (%)	F1 (%)	mAP (%)
YOLOv8n	89.0	88.2	88.6	93.9
PYOLO	95.7	93.7	94.7	98.0

**Fig. 8:**
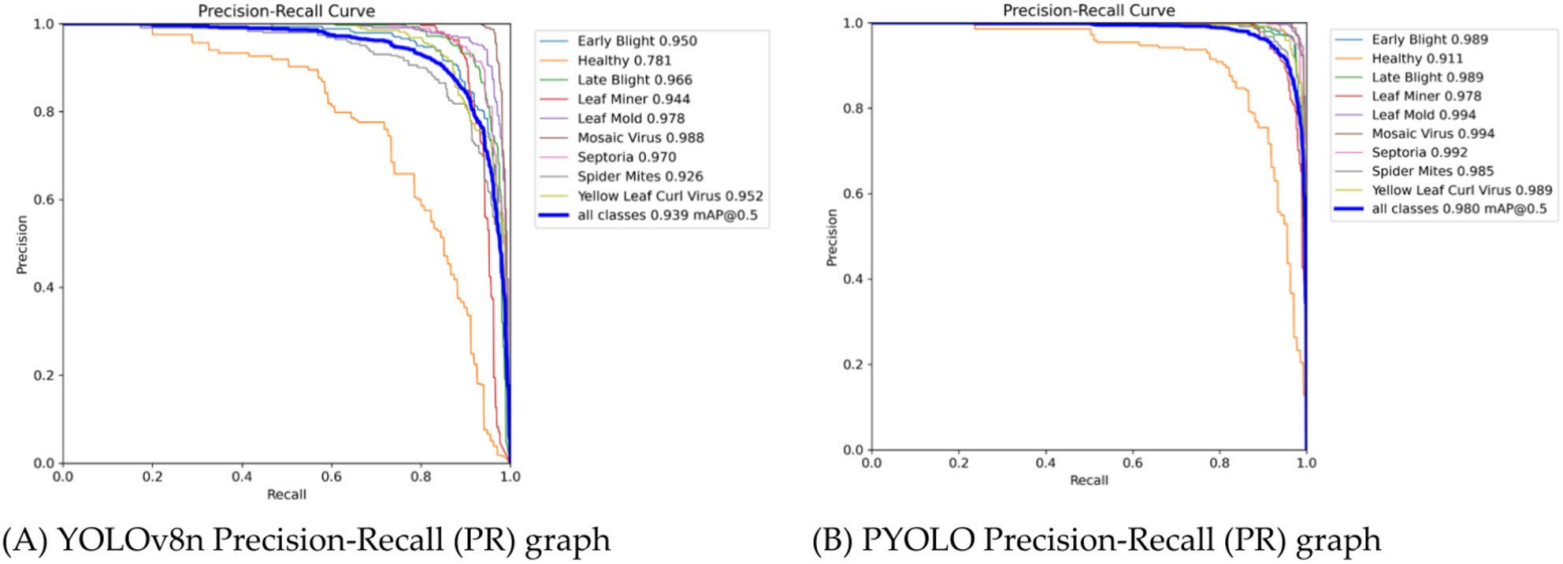
Precision-recall curves comparison. *Note*: (**A**) YOLOv8n PR Graph: Displays the precision-recall curve for the YOLOv8n model, illustrating its performance across various detection thresholds. (**B**) PYOLO PR Graph: Presents the precision-recall curve for the PYOLO model, highlighting its improved detection performance as evidenced by a larger area under the curve (AUC) compared to YOLOv8n.

In the Fig. 8, the area under the curve (AUC), highlighting the model’s performance across different precision and recall settings. A larger AUC indicates better detection performance, confirming PYOLO’s superiority over YOLOv8n.

### Investigative ablation study

To visualize the impact of each module, we conducted ablation experiments, as shown in Table 2. These experiments systematically removed each component of the PYOLO model to evaluate its individual contribution to overall performance. The findings highlight the significance of each module in enhancing detection accuracy and efficiency.

**Table 2:** Results from ablation experiments.

Model	Precision (%)	Recall (%)	F1 (%)	mAP (%)
YOLOv8n	89.0	88.2	88.6	93.9
YOLOv8n + EC2f	90.8	90.6	90.7	95.8
YOLOv8n + EC2f + MHC2f	93.2	92.3	92.7	97.1
PYOLO	95.7	93.7	94.7	98.0

**Fig. 9:**
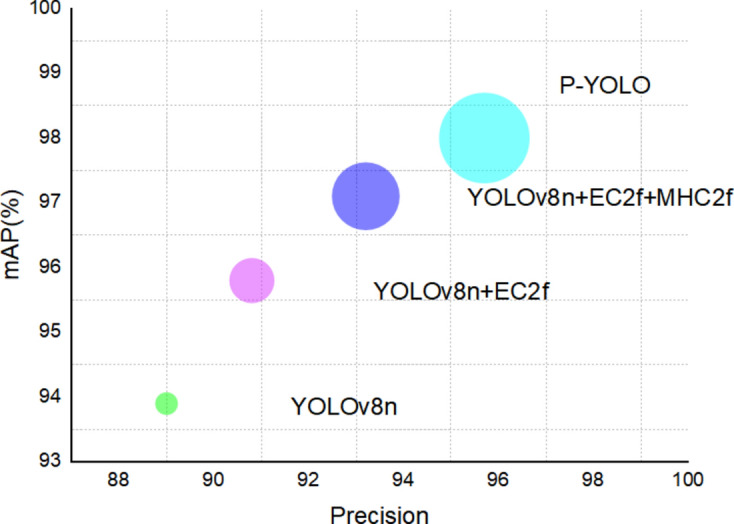
Comparative analysis of mAP across precision.

Table 2 and Fig. 9 revealed that integrating the EC2f module significantly improved our model’s performance: Precision increased by 1.8%, Recall by 2.4%, F1 score by 2.1%, and mAP by 1.9%. These improvements are attributed to the EC2f module’s ability to integrate information from different spatial locations through a convolutional layer, which facilitates spatial feature fusion and improves the diversity and accuracy of feature representations. These results highlight that the EC2f module significantly enhances plant disease detection through advanced spatial feature integration and characterization.

The integration of the MHC2f module improves mean Average Precision (mAP) by 1.3%, thanks to the incorporation of Multihead Self-Attention (MHSA)., which bolsters the model’s ability to discern relationships between different spatial locations and enhances its global information processing capabilities. MHSA enriches the feature representation, resulting in more efficient and accurate performance, particularly in complex scenarios and with diverse target types.

Additionally, the inclusion of the BiFPN module further increases the mAP value by 0.9%. The BiFPN module plays a crucial role in improving information flow between features at different scales. It achieves this by employing a bidirectional feature fusion strategy, which strengthens the connection between low-level and high-level features. This enhancement significantly improves the model’s capability to manage targets of varying scales, thereby making it more robust and effective in a wide range of detection tasks.

Overall, these improvements contribute to a more comprehensive and powerful detection model. The combined effect of the MHC2f and BiFPN modules enables the model to achieve superior accuracy and reliability, ensuring precise plant disease detection even in challenging conditions. This advanced model architecture not only overcomes previous limitations but also establishes a new performance benchmark in plant disease detection.

### Comparative performance of different architectures

To evaluate the PYOLO model’s effectiveness in plant disease detection, comparative experiments with other established models were conducted. Table 3 presents the detailed results.

**Table 3:** Comparative analysis across different models.

Model	Precision (%)	Recall (%)	F1 (%)	mAP (%)
Faster-RCNN	80.4	78.9	79.6	84.8
SSD	82.2	80.5	81.3	86.5
YOLOv3	83.8	82.1	82.9	88.2
YOLOv5	85.6	84.3	84.9	89.7
YOLOv7	87.3	86.5	86.9	91.5
PYOLO	95.7	93.7	94.7	98.0

**Fig. 10:**
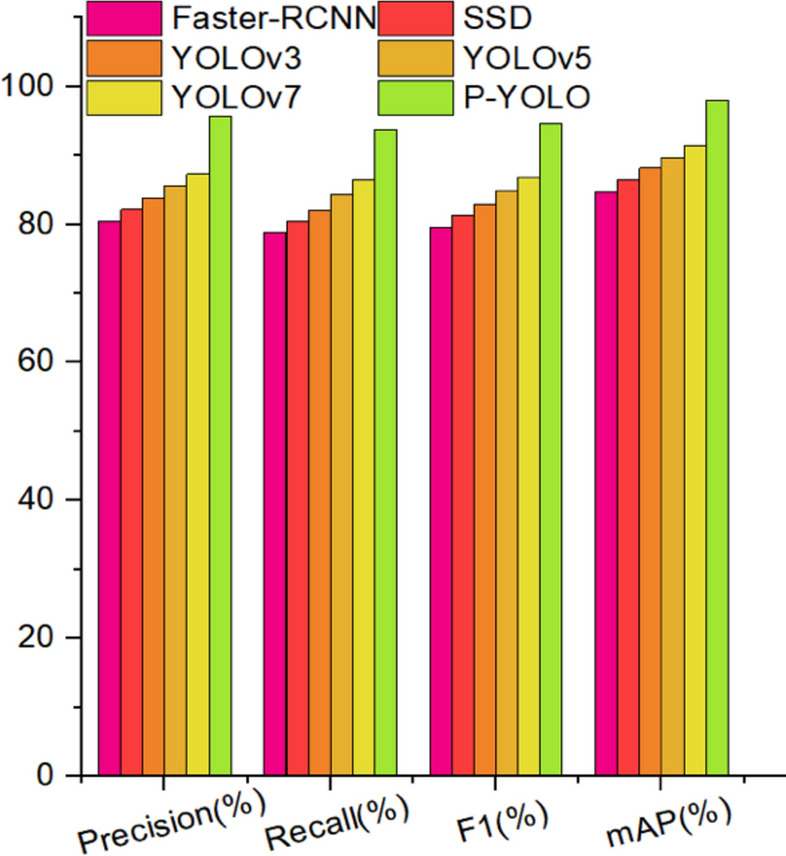
Comparative analysis across different models.

Table 3 exhibited that the Faster-RCNN model has lower detection precision and mean Average Precision (mAP) in plant disease detection, at 80.4% and 84.8%, respectively. This is primarily because the Faster-RCNN model first generates candidate regions of interest (ROIs) and then classifies and regresses these regions, leading to higher model complexity. This complexity reduces its effectiveness in detecting subtle diseases in complex contexts compared to other two-stage models. In contrast, the SSD model improves mAP by 1.7% over Faster-RCNN. SSD uses VGGNet as its base network and makes predictions on multiple feature layers through convolutional layers, resulting in a more concise network structure and higher detection accuracy. However, YOLOv3, a one-stage model, typically achieves faster detection than two-stage models like Faster-RCNN and SSD. YOLOv3 predicts bounding boxes and category probabilities directly within a single convolutional neural network, enhancing detection performance. The YOLOv5 improves upon YOLOv3 by using CSPNet (Cross Stage Partial Networks) as its backbone, which preserves feature diversity and makes the model smaller and more computationally efficient. The mAP of YOLOv7 is 1.8% higher than YOLOv5, due to faster convolutional operations, smaller model size, and the ability to detect more fine-grained objects, thus improving computational efficiency and detection accuracy. Overall, the PYOLO model outperforms these alternatives, demonstrating superior detection capabilities crucial for effective plant disease detection. To objectively illustrate the model’s improvement, we analyzed specific datasets (Fig. 10).

**Fig. 11:**
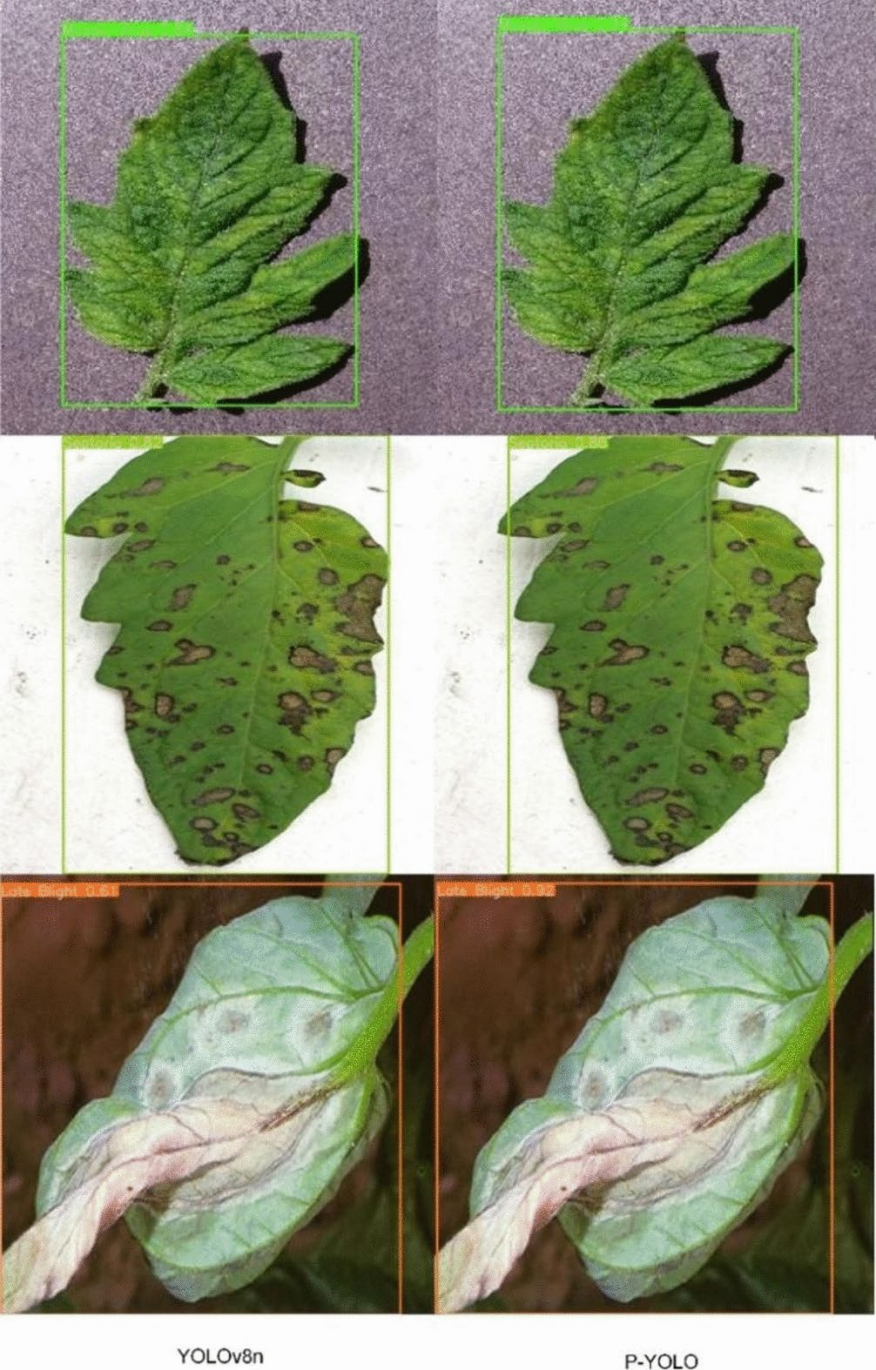
Presentation of experimental findings.

To objectively demonstrate the model’s improvement, we analyzed specific datasets. Figure 11 displayed that the PYOLO model is not only more reliable but also more effective in detecting plant diseases, which is essential for saving crops and reducing economic losses.

## Discussion

Timely and accurate detection of plant diseases is paramount for safeguarding agricultural productivity and mitigating economic losses. This study introduces the PYOLO model, which significantly enhances the capabilities of the YOLOv8n model by addressing critical challenges such as inadequate target feature extraction, variability in target sizes, and difficulties in detecting targets against complex backgrounds.

The PYOLO model enhances the YOLOv8n model in several ways. Firstly, the integration of the EC2f module leverages the channel attention mechanism to emphasize key features and suppress secondary ones, improving feature representation and detection accuracy. Secondly, the BiFPN module enhances detection capability across different scales through cross-scale connectivity and dynamic weight adjustment. Additionally, the MHC2f module, with its parallel processing self-attention mechanism, refines the model’s focus on different image segments, effectively capturing important details in complex scenes.

The PYOLO model introduces several new improvements, especially with the integration of the EC2f module, which uses an advanced channel attention mechanism^39^. This allows for more accurate prioritization of key features while discarding less important ones, thus refining the overall picture. The combination of this system can significantly increase the accuracy of diagnosis, especially in cases where disease symptoms may be subtle or masked by complex plant structures. The implications are profound, as it shows that the PYOLO model not only better identifies obvious symptoms, but also performs well in scenarios where conventional models might fail.

The introduction of BiFPN modules is another important innovation that addresses the challenge of detecting plant disease symptoms that appear at different scales^44^. The PYOLO model significantly increases object detection capabilities across a variety of scales by improving connectivity between scales and enabling dynamic weight adjustment^45^. This is especially important in agricultural situations where diseases can occur in small isolated areas or over large areas. The PYOLO model can dynamically adapt to changes in scale, increasing its applicability in different agricultural situations and making it a powerful tool for real-world implementation.

In addition, the MHC2f module plays an important role in drawing the model’s attention to different parts of the image through a parallel self-attention processing mechanism. This is particularly useful for detecting plant diseases where symptoms may be correlated with other factors or obscured by a complex context. By allowing the model to capture important details in these complex environments, the accuracy of the PYOLO model is greatly improved compared to previous methods. This feature not only improves the detection performance, but also the robustness of the model in real-world applications where environmental factors often create significant noise.

Despite these advancements, the integration of the MHC2f and EC2f modules introduces increased model complexity, necessitating meticulous parameter tuning and sophisticated processing techniques to achieve and maintain optimal performance. Furthermore, the limited size of the current database poses a challenge for generalizing the model across diverse conditions. This underscores the importance of expanding databases to encompass a broader range of plant diseases, environmental conditions, and plant growth stages. Such an expansion would not only enhance the model’s generalizability but also significantly improve its reliability and applicability in diverse real-world scenarios.

Future efforts will concentrate on optimizing the detection model to further elevate plant disease detection performance and adaptability across various scales. Follow-up research will focus on two main areas, including, Applying More Efficient Optimization Algorithms and Learning Rate Scheduling Strategies^46^. These improvements aim to enhance training stability and convergence speed, ensuring that the model can be trained more effectively and quickly, which will involve exploring advanced optimization techniques and fine-tuning learning rates to achieve the best performance. This involves delving into advanced optimization techniques and fine-tuning learning rates to achieve optimal performance. Meanwhile, utilizing transfer learning and pre-trained models will reduce training time and data requirements. This approach allows the model to benefit from previously learned features and patterns, accelerating the training process and improving efficiency.

In addition to these technological developments, future research should consider the broader implications of using advanced measurement systems in real-life agricultural settings^47^. The integration of IoT devices and cloud computing platforms can facilitate real-time monitoring and analysis, potentially leading to greater disease prevention. In addition, the development of user-friendly interfaces and decision support systems will enable farmers to make decisions based on predictive models, making the effective use of technology.

The success of the PYOLO model in improving plant disease detection demonstrates the potential of deep learning to address critical agricultural problems. However, the journey to realize this potential is ongoing and requires innovation and adaptation to the ever-changing environment of technological advances and agricultural needs.

Thus, the PYOLO model is a significant advance in the diagnosis of plant diseases. Its innovative architecture and excellent performance offer broad prospects for further research and applications. Building on these fundamental advances, the agricultural community can look forward to more powerful, efficient and accurate tools to protect crop health and productivity.

## Conclusions

This study proposes an innovative plant disease model called PYOLO. This model integrates the BiFPN network and the newly designed MHC2f mechanism to enhance feature fusion for targets at various scales. Additionally, the introduction of the EC2f mechanism further improves feature learning efficiency across different image subspaces, leading to more detailed and deeper feature extraction. These advancements result in the PYOLO model’s superior sensitivity in plant disease detection. Experimental results confirm the PYOLO model’s potential in effectively identifying and localizing plant diseases. However, there is still potential for optimizing the model structure, particularly in reducing memory usage and improving computational efficiency. Future research will focus on enhancing the model’s generalization by incorporating multi-source datasets, ensuring its comprehensiveness and applicability. Additionally, more efficient network architecture designs, such as improved convolutional layers and activation functions, will be explored to reduce computational complexity and optimize memory usage. Advanced regularization and optimization strategies will also be considered to refine the learning process.

## Data Availability

The improvements and execution process of the YOLOv8 code discussed in this article are available on GitHub, and can be downloaded from https://github.com/WANG9711/my-source-code/releases/tag/yolov8 under the file name yolov8.zip.
